# Efficient Vacuum Deposited P-I-N Perovskite Solar Cells by Front Contact Optimization

**DOI:** 10.3389/fchem.2019.00936

**Published:** 2020-01-17

**Authors:** Azin Babaei, Kassio P. S. Zanoni, Lidón Gil-Escrig, Daniel Pérez-del-Rey, Pablo P. Boix, Michele Sessolo, Henk J. Bolink

**Affiliations:** ^1^Instituto de Ciencia Molecular, Universidad de Valencia, Paterna, Spain; ^2^Helmholtz-Zentrum Berlin für Materialien und Energie GmbH, Berlin, Germany

**Keywords:** perovskite solar cell, molybdenum oxide, vacuum-deposition, processing, hole transport layer

## Abstract

Hole transport layers (HTLs) are of fundamental importance in perovskite solar cells (PSCs), as they must ensure an efficient and selective hole extraction, and ohmic charge transfer to the corresponding electrodes. In p-i-n solar cells, the ITO/HTL is usually not ohmic, and an additional interlayer such as MoO_3_ is usually placed in between the two materials by vacuum sublimation. In this work, we evaluated the properties of the MoO_3_/TaTm (TaTm is the HTL N4,N4,N4″,N4″-tetra([1,1′-biphenyl]-4-yl)-[1,1′:4′,1″-terphenyl]-4,4″-diamine) hole extraction interface by selectively annealing either MoO_3_ (prior to the deposition of TaTm) or the bilayer MoO_3_/TaTm (without pre-treatment on the MoO_3_), at temperature ranging from 60 to 200°C. We then used these p-contacts for the fabrication of a large batch of fully vacuum deposited PSCs, using methylammonium lead iodide as the active layer. We show that annealing the MoO_3_/TaTm bilayers at high temperature is crucial to obtain high rectification with low non-radiative recombination, due to an increase of the electrode work function and the formation of an ohmic interface with TaTm.

Perovskite solar cells (PSCs) are at the forefront of emerging photovoltaics materials, as demonstrated by the continuously rising power conversion efficiency (PCE) (Green et al., 2019). Achieving high conversion efficiencies requires placing the perovskite absorber in between selective charge transport layers that direct charge carriers to the appropriate electrodes for extraction (Pham et al., 2019; Shin et al., 2019). The choice of suitable charge selective materials depends on the type of device architecture used, the particular perovskite absorber, and on the thin-film processing technique. PSCs can be prepared in different architectures, depending on the polarity of the transparent electrode, normally a transparent conductive oxide (TCO) coated on a glass substrate. If the TCO front electrode is used as the p-contact, the structure is referred to as p-i-n, while if electrons are collected at the front contact, the solar cell is termed n-i-p. In p-i-n solar cells, the most common TCO is indium tin oxide (ITO), which is coated with a suitable hole transport layer (HTL) to selectively shuttle holes from the perovskite to the electrode. The hole transport material (HTM) should have a highest occupied molecular orbital (HOMO) or valence band close in energy to the perovskite valence band, as any mismatch would introduce losses in charge extraction or recombination (Schulz et al., 2015). Common molecular HTMs are arylamine derivatives (either polymers or isolated “small” molecules) and polythiophenes, due to their favorable hole mobility and suitable energy level alignment with most perovskite absorbers (Pham et al., 2019). While in some circumstances the direct ITO/HTL p-contact can lead to very efficient charge collection (Al-Ashouri et al., 2019; Liu et al., 2019), the interface is not ohmic, and an additional interlayer is usually placed in between the ITO and the HTL (Schloemer et al., 2019). Common interface materials are high work function molecules (Avila et al., 2018), doped organic semiconductors (Momblona et al., 2016; Schloemer et al., 2019), or metal oxides such as MoO_3_, V_2_O_5_, and W_2_O_3_ (Shin et al., 2019). MoO_3_ is widely adopted as it can be deposited in thin-films by simple thermal vacuum sublimation, resulting in quasi-ohmic interfaces (Schulz et al., 2016). The use of vacuum deposition for transport layers and in particular for the perovskite gives several advantages over solution techniques. The thickness of each layer can be finely controlled, the materials purity is enhanced, and, more importantly, vacuum deposition methods are intrinsically additive, meaning that multilayer devices can be prepared without issues such as intermixing or partial redissolution of materials which are common to solution processing (Ávila et al., 2017). Recently, we have demonstrated the first vacuum-deposited PSCs with metal oxides at both the electron and the hole transporting layers (ETL and HTL) (Pérez-del-Rey et al., 2019). Both the p-i-n or n-i-p structures used MoO_3_ at the p-contact, in combination with N4,N4,N4″,N4″-tetra([1,1′-biphenyl]-4-yl)-[1,1′:4′,1″-terphenyl]-4,4″-diamine (TaTm, Figure 1) as the HTM. In this work, we study the MoO_3_/TaTm interface and use post-deposition treatments in order to ensure an ohmic contact at the interface, demonstrating that a high work function MoO_3_ is required to obtain high efficiency, vacuum deposited p-i-n PSCs.

**Figure 1:**
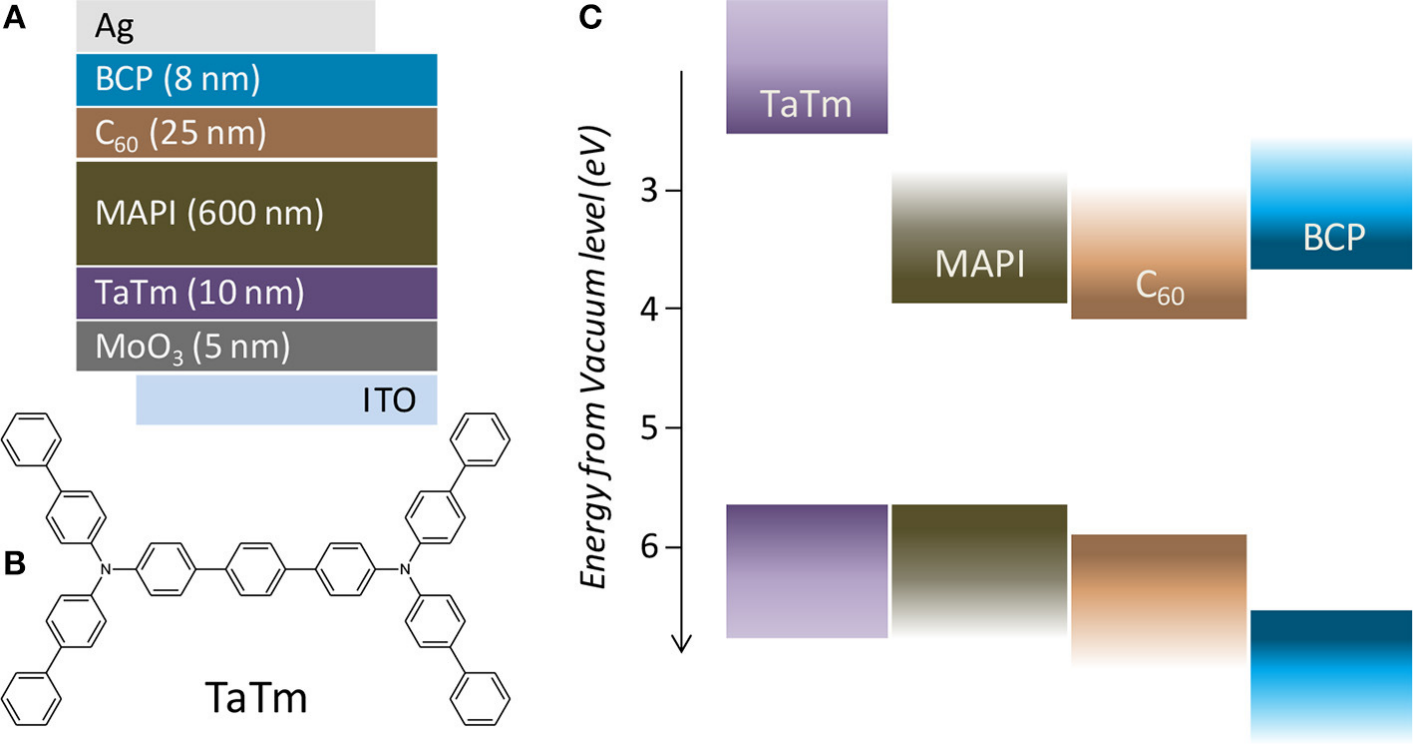
**(A)** Schematics of the device architecture, **(B)** chemical structure of the hole transport layer TaTm and **(C)** flat band energy diagram with the semiconducting materials used in the solar cells.

For this study we used the following device configuration (Figure 1): ITO/MoO_3_ (5 nm)/TaTm (10 nm)/MAPI (600 nm)/C_60_ (25 nm)/BCP (8 nm)/Ag (in which C_60_ is fullerene; BCP is bathocuproine and MAPI is methylammonium lead iodide). TaTm and C_60_ are intrinsic organic materials for charge selection, and MoO_3_ and BCP are p- and n-contacts for efficient extraction of the photogenerated holes and electrons, respectively (Pérez-del-Rey et al., 2019; Zanoni et al., 2019). All the layers in the device, including the MAPI perovskite film, were deposited by vacuum-assisted thermal evaporation, as described in the experimental section in the Supporting Information. To ensure sufficient statistics, for each device configuration, at least two different substrates each containing four cells were evaluated, while for top performing configurations at least five different substrates with a total of 20 cells were characterized. Due to the limited number of substrates (5) that can be processed in the setup used for these experiments, samples were fabricated through several perovskite deposition runs. Hence, small batch-to-batch variations might also contribute to the deviations observed in the photovoltaic parameters. We evaluated the properties of the MoO_3_/TaTm hole extraction interface by selectively annealing either MoO_3_ (prior to the deposition of TaTm) or the bilayer MoO_3_/TaTm (without pre-treatment on the MoO_3_), at temperature ranging from 60 to 200°C in a nitrogen atmosphere for 10 min. We then used these p-contacts for the fabrication of solar cells as described above. The series of devices was initially characterized under simulated solar illumination by measuring the current density vs. voltage (J-V) curves and extracting the relevant PV performance parameters (Figure 2).

**Figure 2:**
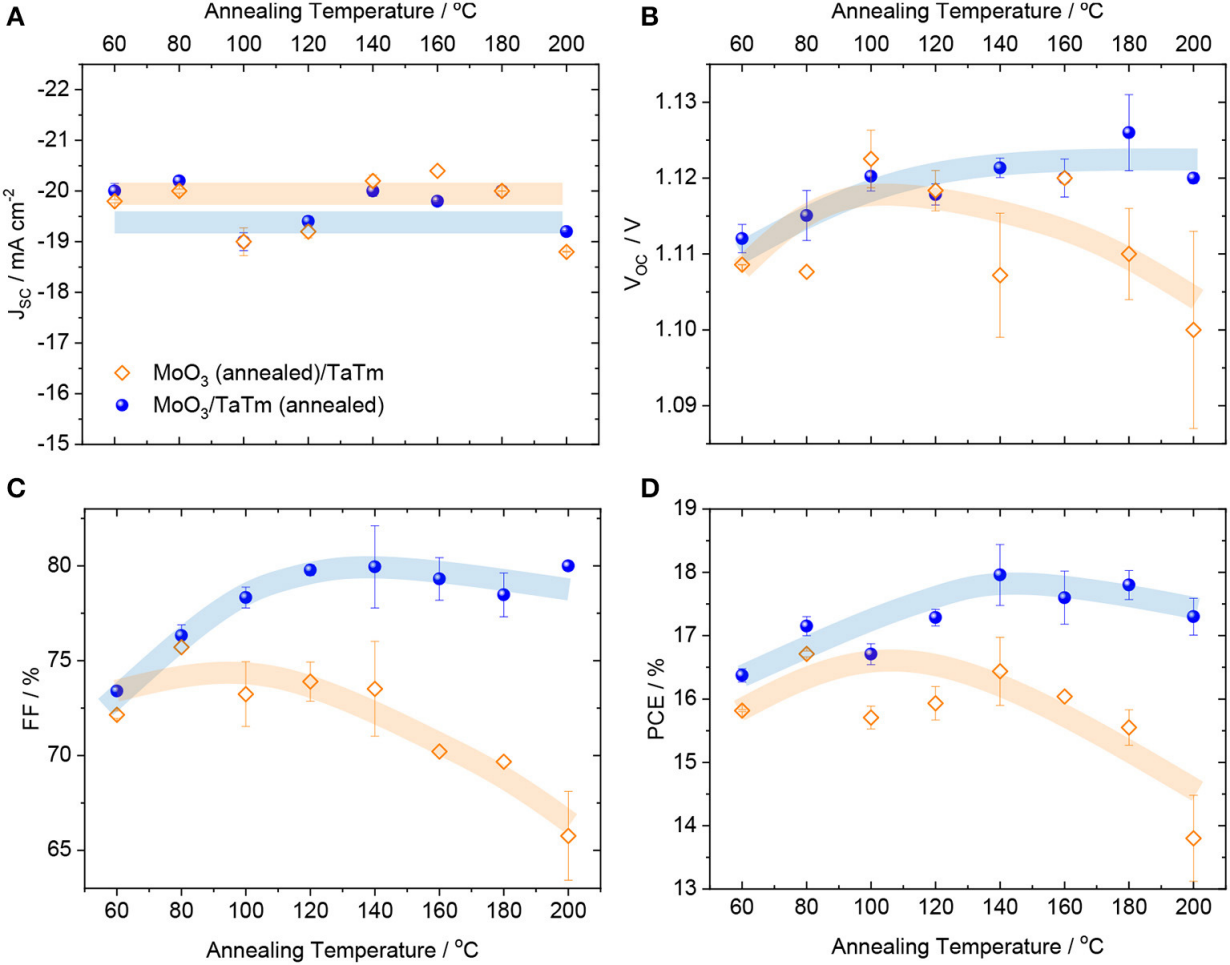
Photovoltaic parameters—**(A)** J_SC_, **(B)** V_OC_, **(C)** FF and **(D)** PCE—extracted form J-V curves under simulated solar illumination, for MoO_3_/TaTm contacts annealed at different temperatures. The blue symbols indicate that the annealing was carried out after deposition of the MoO_3_/TaTm bilayer, while orange symbols correspond to devices where the annealing was carried out on the MoO_3_ prior to depositing TaTm. Lines are a guide to the eye for the reader.

As depicted in Figure 2A, all devices exhibited similar short-circuit current densities (J_SC_) in the 19–20 mA cm^−2^ range, with small fluctuations originated from common batch-to-batch variability in the MAPI perovskite film properties. A difference in the temperature dependence can be observed for the open-circuit voltage (V_oc_, Figure 2B), but especially for the fill factor (FF, Figure 2C). For both device series, we noted an increase in the photovoltage from 60 to ~100°C, after which the V_oc_ was found to be high and stable (>1.12 V) for the cells with annealed MoO_3_/TaTm bilayer, or lower and progressively decreasing for the cells with annealed MoO_3_ (until <1.11 V). The FF (Figure 2C) was found to steadily increase from 73% (at 60°C) to about 80% (in the 120–200°C range) for devices employing the annealed MoO_3_/TaTm bilayer. An opposite behavior was observed when annealing the MoO_3_ prior to the deposition of TaTm. In this case the FF is rather constant at 72–73% until 140°C, when it starts to progressively decrease reaching values of ~65% at 200°C. The evolution of the V_oc_ and especially of the FF with the temperature determine the overall PCE. For solar cells with annealed MoO_3_/TaTm bilayers, the PCE steadily increases from 60°C (16.5%), reaching a maximum of 18% at 140°C, and only slightly diminishing to still above 17% for higher temperatures. On the other hand, the PCE of devices where annealing is carried out on the bare MoO_3_ peaks at 140°C (at 16.5%), but it is strongly limited at higher temperatures, with values below 14% for p-contact annealed at 200°C.

The representative J-V curves under simulated solar illumination for the two types of device with p-contact annealed at 140°C are reported in Figure 3 (J-V curves for all the other investigated annealing temperatures are reported in Figure S1). The device with a MoO_3_ layer annealed before TaTm deposition exhibited fairly high V_oc_ of 1.105 V and J_sc_ of 20 mA cm^−2^, but with a rather poor FF of 73%. This is caused by the reduced slope of the J-V curve after the maximum power point (>0.8 V), which reveals the presence of a high series resistance in these cells (hindered charge extraction/injection). The lower V_oc_ is likely related to the extraction issue, as charge accumulation at the interface can increase the probability of non-radiative recombination, as well as to the higher leakage current (J-V curves in dark, Figure 3B). On the contrary, having the TaTm top-layer deposited and annealed together with MoO_3_ led to solar cells with high rectification (FF = 80%), meaning that charge carrier extraction and injection are enhanced. This can be clearly seen form the dark J-V curves, where the slope of the diffusion region (0.5–1 V) as well as the current density at 1.2 V outperform those of the other type of devices.

**Figure 3:**
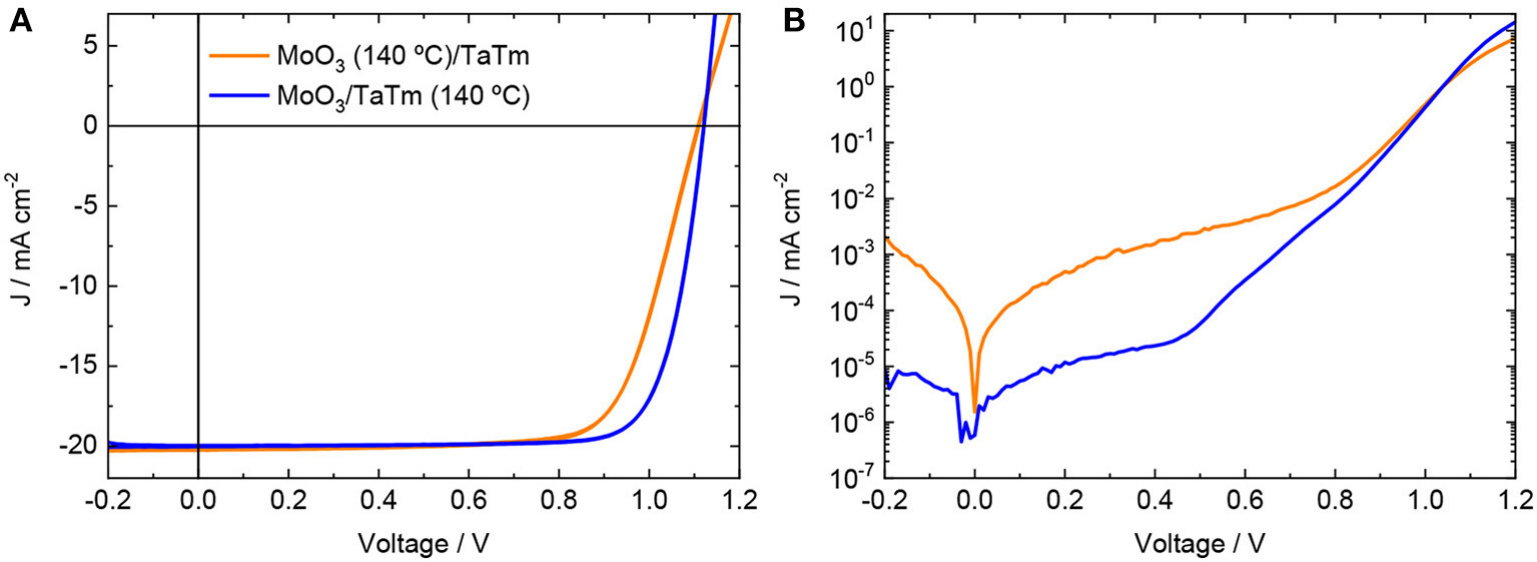
J-V curves **(A)** under simulated solar illumination and **(B)** in the dark for solar cells with structure ITO/MoO_3_/TaTm/MAPbI_3_/C_60_/BCP/Ag, with annealing of the p-contacts performed at 140°C before (orange line) or after (blue line) the deposition of TaTm.

The same set of solar cells with the optimum annealing temperature (140°C) were also studied as function of the incident light intensity, as summarized in Figure 4. The V_oc_ dependence for both devices (Figures 4A,B) showed a higher slope at low light intensity, reduced when approaching 1 sun equivalent illumination (10–100 mW cm^−2^). Fitting the logarithmical dependence of the V_oc_ with the light intensity resulted in diode ideality factors (IF) of 1.9 and 1.4 for the low and high intensity regime, respectively. This behavior can be rationalized on the basis of a dominant trap-assisted recombination (IF = 2) in the bulk of the perovskite at low carrier concentration, and the appearance of surface recombination at the front contact (lowering the IF) which saturates the V_oc_ at higher light intensity (Tress et al., 2018). The trend of intensity-dependent current density for the same devices (Figure S2) also suggest hindered charge extraction (increased recombination) at high light intensity, as the power factor α becomes < 1.

**Figure 4:**
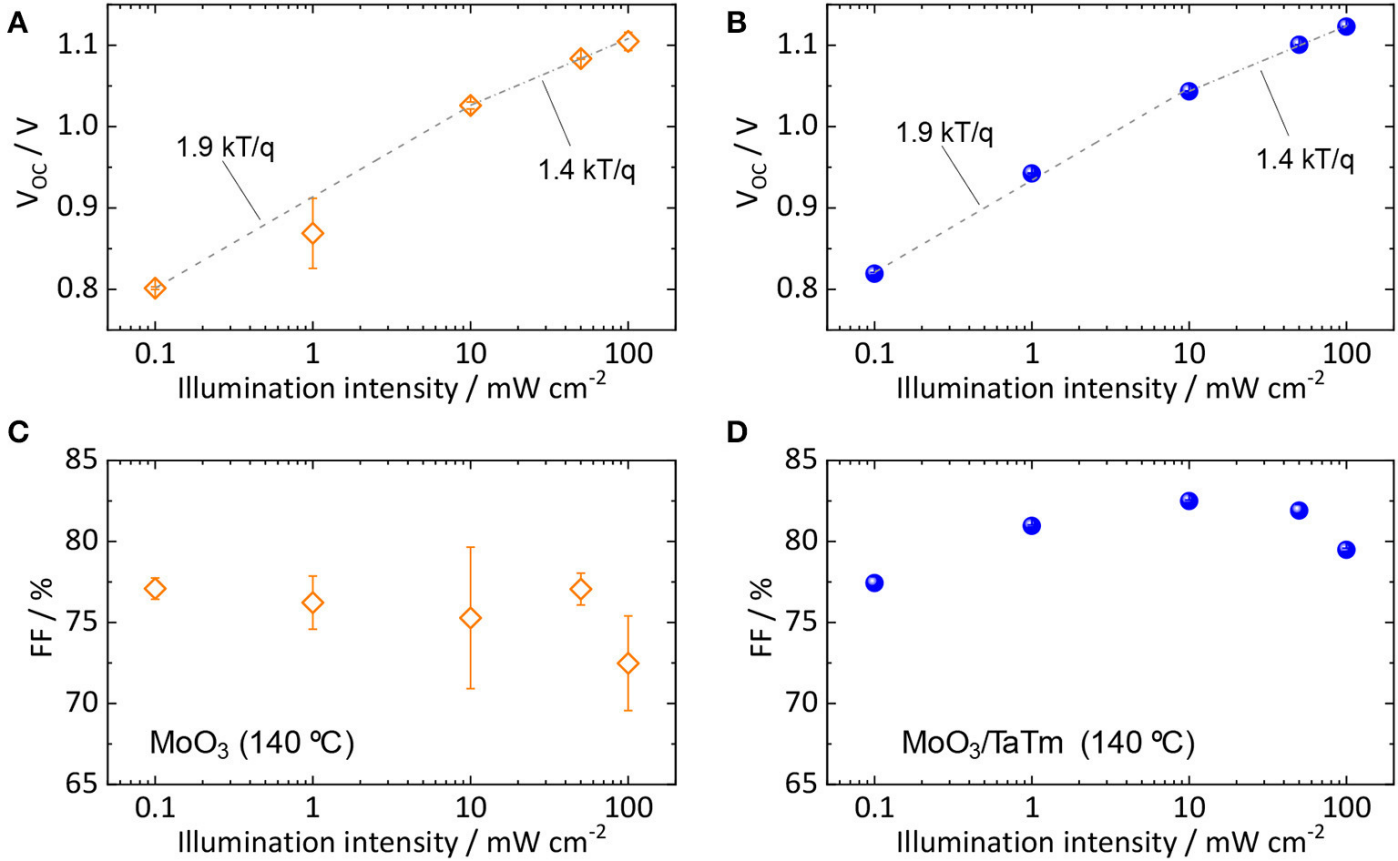
Light intensity dependent characterization of the perovskite solar cells with p-contacts annealed at 140°C. The intensity dependent open-circuit voltage for cells with annealing on **(A)** the bare MoO_3_ and on **(B)** the MoO_3_/TaTm bilayer has been fitted to extract the ideality factor, reported in the graphs. The dependence of the FF for cells employing annealed **(C)** MoO_3_ or **(D)** MoO_3_/TaTm bilayers is also reported.

While no substantial differences could be discerned for the two set of devices, the V_oc_ was found to be systematically larger when annealing the TaTm on top of the MoO_3_ buffer layer, indicating that non-radiative recombination is reduced with this p-contact.

The recombination processes and presence of traps can also be deduced from the trend of the FF with decreasing light-intensity. In a pure trap-assisted recombination regime, the FF decreases with decreasing light intensity as the trap density is constant while the carrier concentration diminishes. For free-carrier recombination, the FF would increase monotonically when decreasing the light intensity, as the recombination rate depends only on the charge densities (Sherkar et al., 2017). As can be seen from Figure 4D for the high efficiency device with annealed MoO_3_/TaTm bilayer, free carrier recombination is present for light intensity above 10 mW cm^−2^, while trap-assisted recombination dominates for low light intensities, in agreement with the trend of photovoltage (Figure 4B). On the other hand, the solar cells with the annealed MoO_3_ did not show a clear trend in the intensity dependence of the FF, remaining rather low (<77%) at all charge carrier concentration. This might indicate the presence of an extraction barrier at the MoO_3_/TaTm interface, which however does not lead to substantial recombination (as the intensity dependent V_oc_ is similar to the other devices). When the majority carriers (holes) are transferred from the perovskite into the TaTm, they can recombine only with the minority carriers (electrons), leading to only small photovoltage losses. However, a potential barrier at the MoO_3_/TaTm interface can still hinder their efficient collection at the front contact, reducing the FF of the solar cells. This barrier would also justify the series resistance observed for these diodes (Figure 3).

In order to shed light on the origin of this phenomena, we performed Kelvin probe measurements on the surface of MoO_3_ as a function of the annealing temperature. We have to note that the experiments were performed in air, and hence the extracted work function (WF) values are affected by spontaneous adsorptions of atmospheric contaminants on the surface of MoO_3_. We observed a WF = 5.05 eV for the sample annealed at 60°C, while for higher temperatures, the MoO_3_ WF was found to decrease of ~0.2 eV (Figure 5). This WF reduction qualitatively agrees with the trend of the FF observed for cells with annealed solely on MoO_3_, presented in Figure 2C. Hence the electrode WF might be held responsible for the extraction barrier and recombination at the front contact. The variation of the WF of MoO_3_ with annealing is likely a consequence of increasing oxygen vacancies (Greiner et al., 2012), as in general reducing the oxidation state of any cationic center (such as by removal of oxygen) tends to decrease the metal oxide WF (Dasgupta et al., 2013). As Kelvin Probe is a surface sensitive technique, we could not extract meaningful information for the MoO_3_ films coated with TaTm. In that case, the loss of oxygen upon annealing might be attenuated by the physical barrier of TaTm itself, leading to a better ohmic contact within the MoO_3_/TaTm interface (Pérez-del-Rey et al., 2019). Additionally, considering the high work function of MoO_3_, hole transfer from the TaTm to MoO_3_ is likely to occur (Xu et al., 2016), resulting in interfacial doping of TaTm and hence beneficial charge extraction.

**Figure 5:**
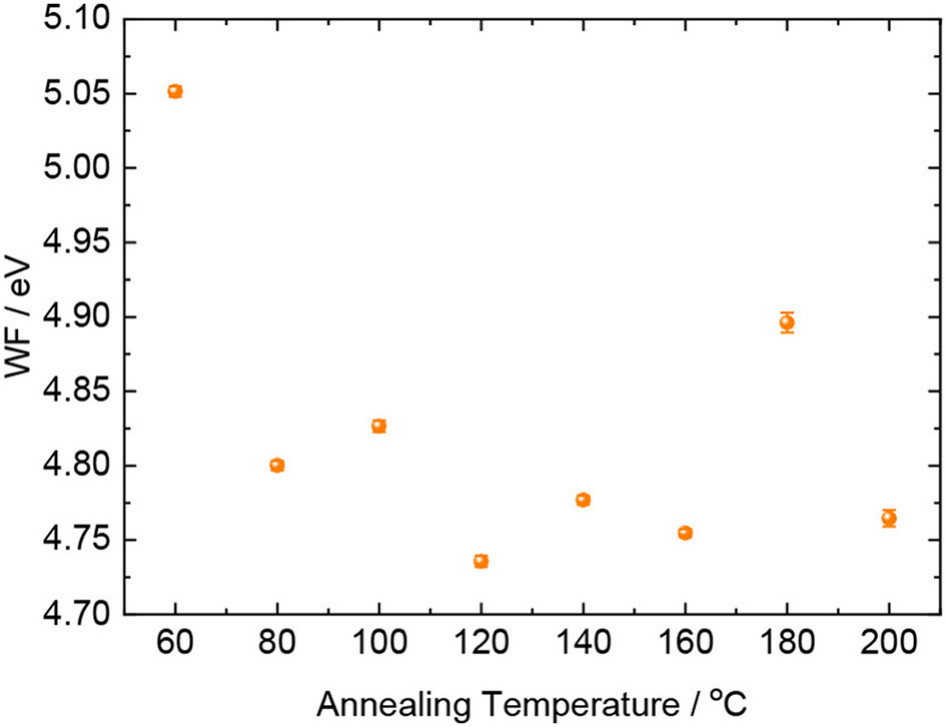
Effect of annealing temperature on the WF of the surface of MoO_3_ thin films deposited on ITO-coated glass slides.

In summary, the power conversion efficiencies of p-i-n PSCs can be modulated through the optimal processing of the MoO_3_/TaTm HTLs. We observed that having the TaTm deposited and annealed together with the MoO_3_ leads to large improvements in fill factor (>80%) and PCE (>18%) at any annealing temperature or light intensity, with the best results obtained for annealing at 140°C. These enhancements are accounted to an improved hole extraction rate and adjusted ohmic contact at the anode, which are likely due to an increased ITO/MoO_3_ electrode work function.

## Data Availability Statement

The datasets generated for this study are available on request to the corresponding author.

## Author Contributions

AB, KZ, and LG-E fabricated and characterized the solar cells. DP-d-R and PB performed part of the characterization. MS and HB conceived the idea and directed the overall project. AB, KZ, and MS wrote the manuscript. All authors read and commented on the manuscript.

### Conflict of Interest

The authors declare that the research was conducted in the absence of any commercial or financial relationships that could be construed as a potential conflict of interest.
