# Supplementary material for: Efficient Vacuum Deposited P-I-N Perovskite Solar Cells by Front Contact Optimization
Source: Front Chem. 2020 Jan 17;7:936. doi: 10.3389/fchem.2019.00936 (PMC6988831; doi:10.3389/fchem.2019.00936)
Supplement: Supplementary file 1 [file Table_1.DOCX]

SUPPORTING INFORMATION

Efficient vacuum deposited p-i-n perovskite solar cells by front contact optimization

Azin Babaei ^1^, Kassio P. S. Zanoni ^1*^, Lidón Gil-Escrig ^2^, Daniel Pérez-del-Rey ^1^, Pablo P. Boix, Michele Sessolo ^1^* and Henk J. Bolink ^1^

*^1^ Instituto de Ciencia Molecular, Universidad de Valencia, C/ Catedrático J. Beltrán 2, 46980 Paterna, Spain*

*^2^ Helmholtz-Zentrum Berlin für Materialien und Energie GmbH, Kekulestraße 5, 12489 Berlin, Germany*

*Corresponding authors: zanoni@ifsc.usp.br; michele.sessolo@uv.es*

**Experimental Section**

Photolithographically patterned ITO coated glass substrates were purchased from Naranjo Substrates. N4,N4,N4′,N4′-tetra([1,1′- biphenyl]-4-yl)-[1,1′:4′,1′-terphenyl]-4,4′-diamine (TaTm) was provided by Novaled GmbH and Fullerene (C_60_) was purchased from Sigma Aldrich. PbI_2_, CH_3_NH_3_I (MAI), MoO_3_, and bathocuproine (BCP) were purchased from Lumtec.

*Device Fabrication.* ITO prepatterned substrates were cleaned following a standard procedure in which they are sequentially cleaned with soap, water, deionized water, and isopropanol in a sonication bath, followed by UV treatment for 20 min. All the solar cell layers were prepared by thermal vacuum deposition performed in vacuum chambers evacuated to a pressure of 10^−6^ mbar, which were integrated into a nitrogen-filled glovebox (H_2_O and O_2_ < 0.1 ppm). In general, the vacuum chambers were equipped with temperature controlled evaporation sources (Creaphys) fitted with ceramic crucibles. The sources were directed upward with an angle of approximately 90° with respect to the bottom of the evaporator. The distance between the substrate holder and the evaporation source was approximately 20 cm. Individual quartz crystal microbalance (QCM) sensors monitored the deposition rate of each evaporation source and another one close to the substrate holder monitored the total deposition rate. TaTm, MAPbI_3_, C_60_ and BCP were sublimed in the same vacuum chamber at temperatures ranging from 60 to >300 °C, depending on the material, and the precise evaporation rate and deposited film thickness were controlled by the QCM sensors. In general, the deposition rate for TaTm and C_60_ was 0.5 Å s^−1^ while the thinner BCP layer was evaporated at 0.2−0.3 Å s^−1^. For the perovskite deposition, MAI and PbI_2_ were co-evaporated at the same time by measuring the deposition rate of each material in two different sensors (with rates of 1.0 and 0.6 Å s^−1^, respectively) and obtaining the total perovskite thickness in the third one, leading to a 600 nm-thick perovskite. MoO_3_ and Ag were evaporated in another vacuum chamber using aluminum boats as sources by applying currents ranging from 2.0 to 4.5 A.

*General characterizations.* For the solar cell characterization, the J−V curves were recorded using a Keithley 2612A SourceMeter in a −0.2 and 1.2 V voltage range, with 0.01 V steps and integrating the signal for 20 ms after a 10 ms delay, corresponding to a speed of about 0.3 V s^−1^. The devices were illuminated under a Wavelabs Sinus 70 LED solar simulator. The light intensity was calibrated before every measurement using a calibrated Si reference diode equipped with an infrared cut-off filter (KG-3, Schott). Intensity dependent measurements where carried out by collecting J-V curves with different neutral density filters of decreasing optical density. The layout used to test the solar cells has four equal areas (0.083 cm^2^, defined as the overlap between the ITO and the top metal contact) and measured through a shadow mask with 0.050 cm^2^ aperture. The work functions were determined by Kelvin probe measurements using an Ambient Pressure Photoemission Spectroscopy system from KP Technology.


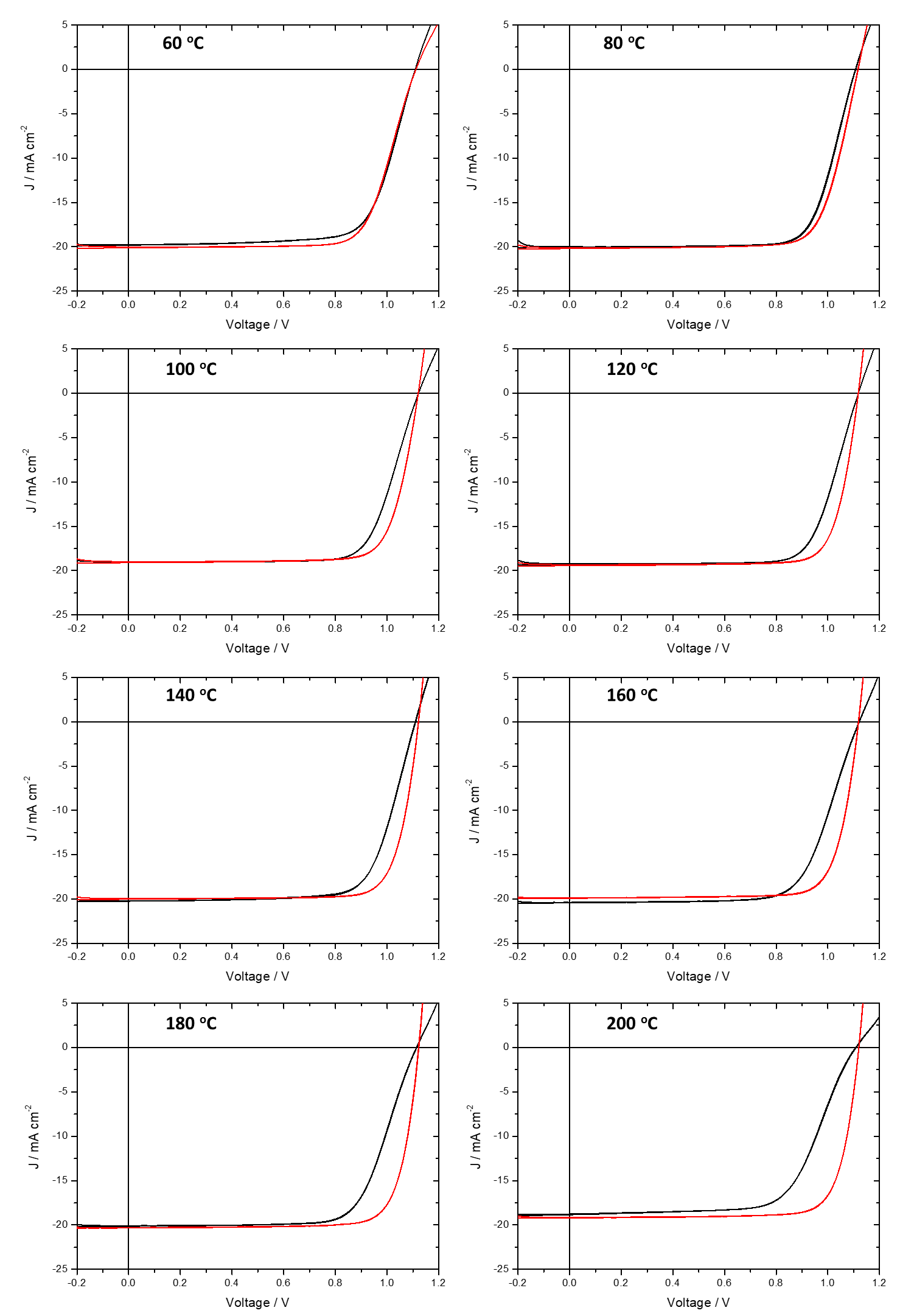


Figure S1. Illuminated JV curves of ITO/MoO_3_/TaTm/MAPbI_3_/C_60_/BCP/Ag solar cells, with annealing of MoO_3_ at different temperatures performed before (black line) or after (red line) the deposition of TaTm.

Figure S2. Intensity dependent photocurrent of the perovskite solar cells with p-contacts annealed at 140 ºC. The intensity dependent short-circuit current densities for cells with annealing on (A) the bare MoO_3_ and on (B) the MoO_3_/TaTm bilayer has been fitted with a power law J ∝ I^α^, where I is the incident light intensity.
